# Effect of enclosure on reproductive allocation of wheatgrass *Agropyron mongolicum* populations in desert steppes

**DOI:** 10.1002/ece3.5839

**Published:** 2019-11-19

**Authors:** Guoqi Li, Panpan Zhao, Wenshan Shao, Changqing Jin, Lixiao Song, Yanyun Chen

**Affiliations:** ^1^ Breeding Base for State Key Laboratory of Land Degradation and Ecological Restoration in Northwest China Ningxia University Yinchuan China; ^2^ Key Laboratory for Recovery and Restoration of Degraded Ecosystem in North‐western China of Ministry of Education Ningxia University Yinchuan China; ^3^ Forestry Technology Extension Center of Liangzhou District of Wuwei City in Gansu Province Wuwei China

**Keywords:** *Agropyron mongolicum*, desert steppes, enclosure, reproductive allocation

## Abstract

Plants generally adopt different reproductive strategies to adapt to their environments and increase their fitness. Here, we studied the effects of enclosure cultivation on the reproductive allocation of a wheatgrass species *Agropyron mongolicum* in the Desert steppes of Northern China. The results showed that: (a) after enclosure cultivation, the height, clump width, coverage, and clump biomass of *A. mongolicum* significantly increased by 78.96% (*p* = .040), 63.50% (*p* = .013), 50.89% (*p* = .032), and 205.38% (*p* = .022), respectively, whereas density did not show a significant change (*p* = .330). (b) Enclosure cultivation significantly affected the biomass of *A. mongolicum*. Compared with cultivation outside the fence, root, leaf, and spike biomass of *A. mongolicum* inside the fence significantly increased by 183.52% (*p* = .020), 334.09% (*p* = .011), and 381.25% (*p* = .005), respectively. In addition, root biomass was the highest among the components (38.53 and 13.59 g inside and outside the fence, respectively) and spike biomass was the lowest (6.16 and 1.28 g inside and outside the fence, respectively). (c) Enclosure cultivation affected elemental nutrient allocation and the caloric values of various components of *A. mongolicum*, and the caloric values are positively correlated with carbon, nitrogen, and phosphorus contents. Enclosure cultivation significantly reduced carbon, nitrogen, and phosphorus in the roots, as well as nitrogen and phosphorus in the spikes, but significantly increased nitrogen in the spikes by 9.78%. The caloric values of *A. mongolicum* inside and outside the fence in decreasing order were as follows: spikes > leaves > stems > roots. Comparison of cultivation effects between inside and outside the fence showed that the caloric values of the spikes and roots significantly increased by 0.92% and 1.60%, respectively, whereas those of the leaves significantly decreased by 0.70%. Our results demonstrate that the reproductive allocation of elemental nutrients and caloric values in nonreproductive and reproductive organs are plastic to arid environments.

## Graphical abstract

Enclosure cultivation affected elemental nutrient allocation and the caloric values of various components of *Agropyron mongolicum*, and the caloric values showed extremely significant positive correlation with carbon, nitrogen, and phosphorus content.

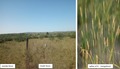

## 1 INTRODUCTION

Plant reproductive allocation refers to the net investment of resources in the reproduction of plants (Karlsson & Méndez, 2005), which can reflect the response and adaptation of plant growth and development to the environment to some extent (Atson, Geber, & Jones, 1995; Li, Li, & Liu, 2004; Schmid, 1990). Plant reproduction traits are associated with specific resource utilization strategies during succession. For example, acquisitive species may invest less energy and resources on reproduction in early succession, while conservative species may invest more energy and resources on reproduction in late succession (Han et al., 2019). In a general sense, specific reproductive allocation strategies result from the self‐organization processes toward optimal resource allocation patterns during the entire life history, which can eventually improve plant fitness (Cheplick, 2005; He & Zhong, 1997).

Desert steppes have harsh environmental conditions that severely limit the growth, development, and reproduction of plants. Such stressful conditions (generally characterized by drought) often give rise to unique reproductive allocation strategies through long‐term adaptation and selection (Zhang et al., 2005). Apart from environmental stress, livestock may also play an important role in shaping plant reproductive allocation strategies in desert steppe ecosystems, for example, grazing affects on biomass and morphology variation (Motamedi, Sheidai Karkaj, & Alilou, 2016), plant diversity and grassland productivity (Zhang et al., 2018), plant community composition (Porensky, Derne, Augustine, & Milchunas, 2016). However, so far, the long‐term effect of livestock on plant reproductive strategies remains elusive due to the lack of experimental evidence.

In this study, we aim at tackling this difficulty using a long‐term enclosure experiment carried out through the “Grain‐for‐Green Project” in China. The Grain‐for‐Green Project is a large‐scale ecological restoration program implemented by the Chinese national government since the year 2000 onwards, aiming at returning cultivated lands to forests or perennial grasslands across the whole China. By the year 2014, through this project, a total degraded area of 29.9 million ha had been successfully restored (Lv, Ma, & Peng, 2019). In drylands, grazing enclosure has been identified as an effective measure of this project for the purpose of restoring degraded rangelands due to overgrazing (Deng, Shangguan, Wu, & Chang, 2017). Our study site is located in the Yanchi County, Ningxia Autonomous Region, Northwestern China, where grazing enclosure (as a part of the national Grain‐for‐Green Project) has been implemented through fencing in a typical temperate desert steppe ecosystem since 2000. All livestock including cattle, sheep and goats are completely removed inside the fences. This implementation of fencing makes a suitable system for studying the long‐term effect of livestock on plant reproduction strategy. Here, we focused on the perennial grass species *Agropyron mongolicum* as the dominant plant species in our study site. Trade‐off between growth and reproduction of plants can be reflected by a range of plant traits including biomass, elemental nutrients and caloric values (Bao et al., 2006; Xu et al., 2014; Zhao, 2007). Our specific aims are to (a) examine changes in module biomass induced by grazing enclosure; (b) compare module elemental nutrients, and caloric values of plants between the conditions with and without fencing; and (c) analyze correlation among biomass, caloric values, and elemental nutrient contents of various components of *A. mongolicum* populations under enclosure cultivation. We hypothesized that the reproductive allocation of *A. mongolicum* will change as a result of the 18‐year grazing enclosure.

## 2 MATERIALS AND METHODS

### 2.1 Study species

**Figure 1:**
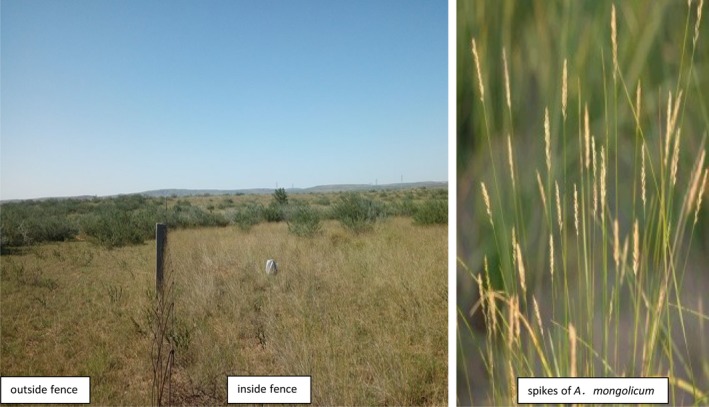
A partial overview of field experimental plots and a photo of *Agropyron mongolicum*

Mongolian wheatgrass *A. mongolicum* is a perennial grass belonging to family Gramineae. This grass species has high adaptability (Zhao, 2004) with strong anti‐drought, anti‐cold, and wind and sand resistance. *Agropyron mongolicum* is often seen in grasslands and desert steppes as an accompanying species (Yun & Mi, 1989) and is a superior forage grass. Figure 1 showed the partial overview of field experimental plots and the spikes of *A. mongolicum*. The perennial grass species *A. mongolicum* and *Stipa capillata* have become the dominant plant species after the 18‐year fencing. The shrub species *Caragana korshinskii* can be occasionally found inside the fences, whereas the shrub species *C. korshinskii* and poisonous plant species *Cynanchum komarovii* are the main species outside the fences (Li, Shao, Zhao, & Jin, 2018).

### 2.2 Overview of study site

**Figure 2:**
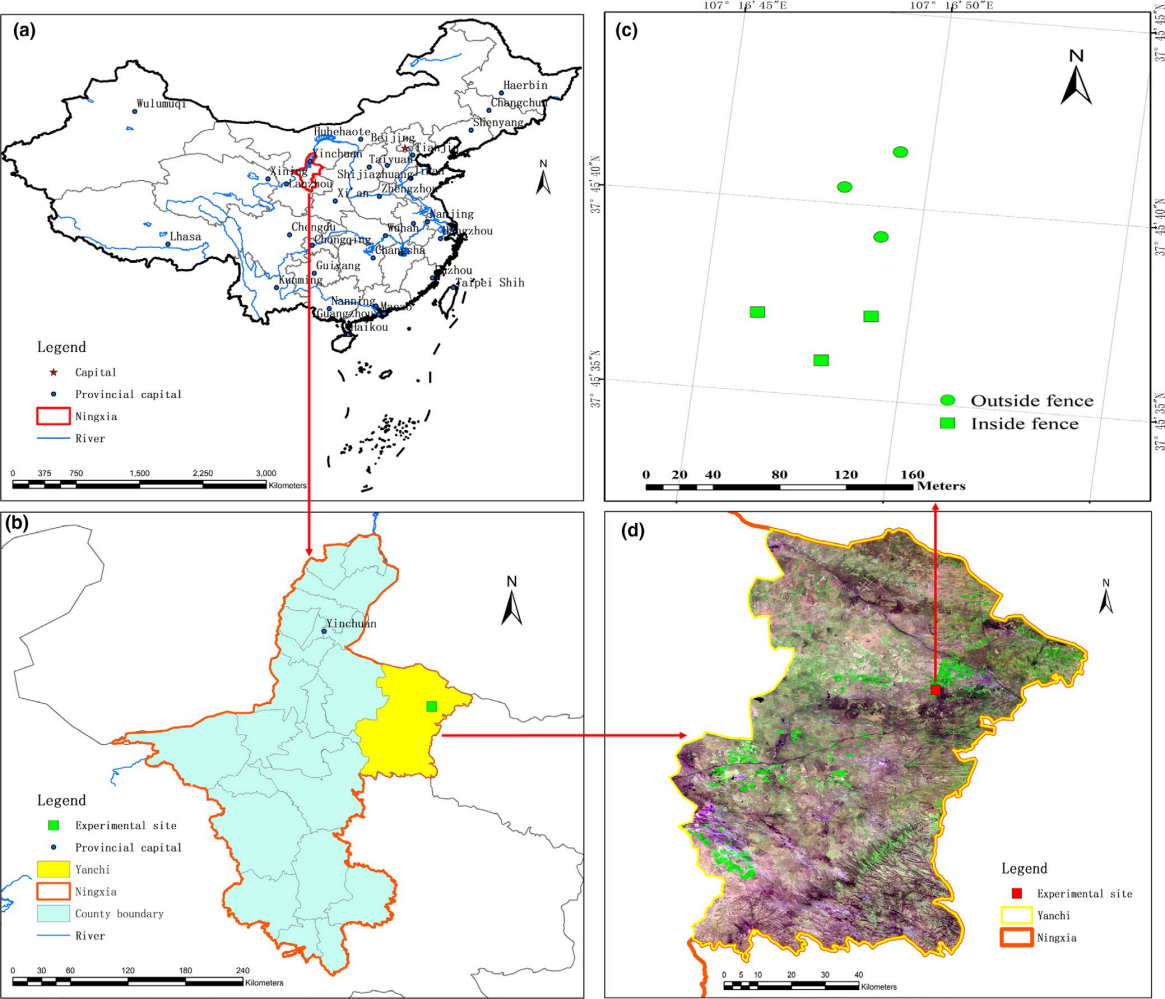
The locations of the sampling sites (a: China, b: Ningxia, c: Yanchi, d: Sampling sites)

The study site is located in the Sidunzi Village of Yanchi County, which is in the eastern part of Ningxia Autonomous Region, China (E106°30′10″–107°48′, N37°04′–38°10′). It is located on the southwestern edge of the Mu Us Sandyland and in a transition region between the Loess Plateau and the Ordos Plateau. The site has adverse natural conditions, and the climate is mid‐temperate continental monsoon climate. The mean annual temperature is 7.7°C, and the mean annual precipitation is 250–350 mm, which is mostly concentrated in June to September. Annual evaporation is around 2,500 mm, and the annual frost‐free period is 165 days. The terrain consists of gently sloping hills (Li et al., 2018). The soil mainly consists of gray calcium soil, aeolian sandy soil, and loessial soil. Soil texture is sandy and silty (Yang, Yang, Wang, & Jia, 2003). The study site is a transition zone in a temperate grassland in China, which comprises the Eurasian steppes. The community shows a mosaic distribution pattern of xerophytic plants and perennial grasses as structural species of typical grasslands. The predominant species of the study site include *A. mongolicum*, *A. scoparia*, *A. scoparia*, *Lespedeza potaninii*, *C. komarovii*, *Stipaca pillata* and *Caragana crororshisrii* (Shao, Li, Chen, & Zhao, 2016). Figure 2 showed the locations of the sampling sites.

### 2.3 Study methods

#### 2.3.1 Sampling

In early August 2017, which is the maturation period for *A. mongolicum* seeds, we randomly selected three 100 m^2^ (10 m × 10 m) large quadrats along diagonal lines in a 16‐year enclosed (enclosure constructed in 2001, total coverage: 65%–75%) and nonenclosed (total coverage of 40%–50%) plots at locations with the same climatic conditions and geographical positions. One 4 m × 4 m medium quadrat is set up at each of the four corners of the large quadrat before five 1 m × 1 m small quadrats were set up in a quincunx. Each sample plot contains 60 small quadrats, making a total of 120 quadrats. The height, coverage, clump width, and density of *A. mongolicum* in each small quadrat were recorded. Then, 30 clumps of *A. mongolicum* were randomly selected, and the clumps were completely dug up to 1 m depth due to deep root, so that the biomass of the entire clump could be obtained. After the collected samples were transported to the laboratory, the aboveground organs were separated into vegetative and reproductive branches. The stems and leaves of the vegetative branches and the stems, leaves, and spikes of the reproductive branches were separated. Weeds and dead roots were removed from the belowground organs before washing the belowground organs. The components were packed in paper bags and dried at 65°C until constant weight and then weighed. The components were crushed, and the caloric values and elemental nutrient contents of the components were measured. The conventional methods were adopted to calculate plants height, clump width, coverage, density and total biomass (Zhang, 2004).

#### 2.3.2 Measurements of nutrients and caloric values

The total carbon, total nitrogen, total phosphorus and caloric values of plant module (roots, stems, leaves, and spikes) were measured in 2017. Total carbon measured by Potassium dichromate volumetric method‐external heating method, total nitrogen measured by semi‐micro Kjeldahl method, total phosphorus measured by Mo‐Sb colorimetric method (Bao, 2000). After air‐drying, fresh samples were ground and passed through a 2 mm sieve, dried at 65°C to constant weight. Caloric values were measured by ZDHW‐6 automatic calorimeter with three replicates (Xu, Zhang, Feng, & Zhang, 2003).

#### 2.3.3 Data analysis

Paired *t* test in ANOVA was used for significance analysis (*α* = .05) on comparing each class between inside and outside fences. Pearson's correlation coefficient analysis was used to determine correlation between three components including biomass, caloric value, and nutrient elements content. SPSS 17.0 was used for data processing. Microsoft Excel 2010 was used for data organization and graph plotting for experimental data.

## 3 RESULTS AND ANALYSIS

### 3.1 Effects of enclosure cultivation on the characteristics of *A. mongolicum* populations

Table 1 shows that after enclosure cultivation, the height, clump width, coverage, and clump biomass of the *A. mongolicum* populations significantly increased by 78.96% (*p* = .040), 63.50% (*p* = .013), 50.89% (*p* = .032), and 205.38% (*p* = .022), respectively. Density did not change (*p* = .330).

**Table 1:** Population characteristics of *Agropyron mongolicum* inside and outside the fence

Item	Inside the fence	Outside the fence
Height (cm)	57.57 ± 3.86**	32.17 ± 1.51
Clump width (cm)	77.17 ± 6.35**	47.20 ± 2.88
Coverage (%)	59.63 ± 2.35**	39.52 ± 1.36
Density (clump/m^2^)	7.73 ± 2.44	6.80 ± 0.81
Total biomass (g/clump)	88.04 ± 7.71**	28.83 ± 1.57

Note

* Significant correlation at the <0.05 level.

** Significant correlation at the <0.01 level.

### 3.2 Effects of enclosure cultivation on the biomass of various components of *A. mongolicum*

**Figure 3:**
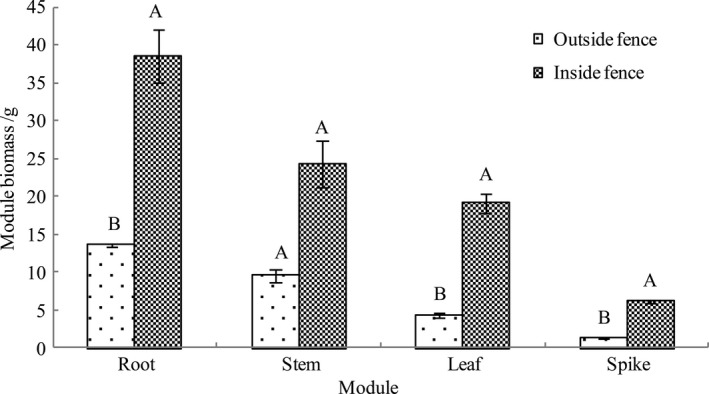
Module biomass of *Agropyron mongolicum* population inside and outside the fence (Mean ± *SE*). Different capital letters indicate the significant difference between two sample plots (*p* < .05, *n* = 30)

Figure 3 shows that the biomass of the roots, stems, leaves, and spikes of *A. mongolicum* grown outside the fence was 13.59, 9.56, 4.40, and 1.28 g, respectively. The biomass of these components in *A. mongolicum* grown inside the fence was 38.53, 24.25, 19.10, 6.16 g, respectively. The biomass of *A. mongolicum* components inside and outside the fence arranged in decreasing order was as follows: roots > stems > leaves > spikes, with root biomass significantly higher than that of the stems, leaves, and spikes. Significant differences in biomass between various components of *A. mongolicum* grown outside the fence were observed (*p* = .0001). For *A. mongolicum* grown inside the fence, significant differences in biomass for all components were detected, except for the stems and leaves (*p* = .021). Compared to *A. mongolicum* grown outside the fence, the biomass of the roots, leaves, and spikes of *A. mongolicum* significantly increased after enclosure cultivation by 183.52% (*p* = .020), 334.09% (*p* = .011), and 381.25% (*p* = .005), respectively, although stem biomass did not change (*p* = .065).

### 3.3 Effects of enclosure cultivation on elemental nutrient content in various components of *A. mongolicum*

**Figure 4:**
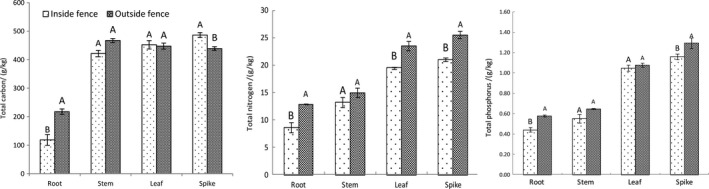
Nutrient allocation for each component of *Agropyron mongolicum* inside and outside the fence

Figure 4 shows the results of a comparison of allocation status of nutrients in various *A. mongolicum* components inside and outside the fence. Inside the fence, the roots showed the lowest total carbon, total nitrogen, and total phosphorus content, whereas the spikes exhibited the highest values. Outside the fence, the roots showed the lowest total carbon, total nitrogen, and total phosphorus content, whereas the spikes exhibited the highest total nitrogen and total phosphorus content. The effects of enclosure cultivation varying among the components of the *A. mongolicum* populations, wherein the total carbon, total nitrogen, and total phosphorus content of the roots, total nitrogen and total phosphorus contents of the spikes, and total nitrogen content of the leaves decreased significantly (*p* < .05). However, the total carbon content of the spikes significantly increased by 9.78%, whereas no significant changes in all elemental nutrients were detected in the stems.

### 3.4 Effects of enclosure cultivation on energy allocation in various components of *A. mongolicum*

Table 2 shows the energy allocation status in various *A. mongolicum* components inside and outside the fence. Overall, the respective caloric values of the roots, stems, leaves, and spikes of *A. mongolicum* grown inside the fence were 17.13, 17.17, 18.52, and 18.82 kJ/g, whereas those of *A. mongolicum* grown outside the fence were 16.86, 17.32, 18.65, and 19.64 kJ/g. *Agropyron mongolicum* grown inside and outside the fence showed an increase in caloric value in this order: spikes > leaves > stems > roots. Compared to *A. mongolicum* grown outside the fence, the caloric values of the spikes and roots after enclosure cultivation significantly increased by 0.91% (*p* = .010) and 1.60% (*p* = .042), respectively, whereas the caloric values of the leaves significantly decreased by 0.70% (*p* = .029), and no significant change was observed in the stems (*p* = .126). These findings indicate that regardless of growth site, the caloric values of all aboveground organs from *A. mongolicum* populations were all larger than that of the underground roots. In addition, the caloric value of the reproductive organs (spikes) extremely significantly increased inside the fence compared to those outside the fence.

**Table 2:** Energy (caloric value) for each component of *Agropyron mongolicum* inside and outside the fence

Plant components	Caloric value Q/(kJ/g)		*p*
	Inside the fence	Outside the fence	
Roots	17.13 ± 0.02	16.86 ± 0.08	.042
Stems	17.17 ± 0.09	17.32 ± 0.07	.126
Leaves	18.52 ± 0.02	18.65 ± 0.01	.029
Spikes	19.82 ± 0.01	19.64 ± 0.03	.010

### 3.5 Analysis of the correlation among biomass, caloric values, and elemental nutrient contents of various components of *A. mongolicum*

Table 3 shows the correlation relationships among biomass, caloric values, and elemental nutrient contents of the various components of *A. mongolicum*. The biomass of various *A. mongolicum* components showed an extremely significant negative correlation with caloric values, as well as total carbon, nitrogen, and phosphorus contents (*p* < .01). Of these correlations, that between biomass and total nitrogen content was the strongest at −0.804 (*p* < .01). A significant positive correlation was observed between the caloric values of various components and the elemental nutrients (*p* < .01), with that of total phosphorus content the strongest at 0.947 (*p* < .01). In addition, a significant positive correlation was detected among various elemental nutrients, with that between total phosphorus and total nitrogen the highest, reaching 0.953 (*p* < .01).

**Table 3:** Pearson's correlation coefficients for each component of *Agropyron mongolicum* biomass, caloric value, and nutrient elements content

Item	Biomass	Caloric value	Total C	Total N	Total P
Biomass	1				
Caloric value	−0.631**	1			
Total C	−0.689**	0.611**	1		
Total N	−0.804**	0.869**	0.716**	1	
Total P	−0.722**	0.947**	0.663**	0.953**	1

* Significant correlation at the <0.05 level.

** Significant correlation at the <0.01 level.

## 4 DISCUSSION

Enclosure cultivation alleviates trampling and feeding by livestock, enabling grassland ecosystems to succeed in a certain direction under natural conditions (Yang et al., 2016). In this study, the height, clump width, coverage, and total biomass of *A. mongolicum* populations significantly increased after enclosure cultivation, whereas changes in density were not significant. These findings indicate that reduced external interference and habitat improvements during enclosure cultivation are favorable for vegetation recovery and increasing productivity. First, as a predominant species, *A. mongolicum* itself is a superior forage grass. Enclosure cultivation prevents *A. mongolicum* from being eaten and trampled on by livestock, thereby effectively increasing the height, clump width, coverage, and total biomass of *A. mongolicum*. Second, soil nutrient accumulation, increased elemental nutrient supply, and improvements in soil physical characteristics after enclosure cultivation facilitate plant growth and proliferation (Hosseinzadeh, Jalilvand, & Tamartash, 2010; Liu, Wang, Chen, Wang, & Han, 2006). These results are consistent with the findings of previous studies (Jin et al., 2017; Lamas, Larreguy, Carrera, & Bertiller, 2013). No significant changes in *A. mongolicum* population density were observed, which may be attributable to the fact that *A. mongolicum* mainly employs asexual reproduction (tillering), and seed reproduction is secondary, and this may be related to the density effect of the species inside the fence. However, livestock feeding and soil fertility affect sexual reproduction of *A. mongolicum* outside the fence.

Biomass is an important marker that reflects plant–environment interactions and is a manifestation of the plant's environmental adaptive capacity and growth and development patterns. In addition, the biomass of individuals and components is also a manifestation of the ability of ecosystems to acquire energy (Yu & Yu, 2001). Under a natural enclosed grassland ecosystem, vegetation responds to various resources through diverse and complex adjustments, thereby effectively increasing the utilization efficiency of limited resources by the entire ecosystem (Hutchings, 1995; Pan, Wang, Jia, Chen, & He, 2008; Wang, Li, Xiao, & Pan, 2006). In this study, root biomass was significantly greater than the biomass of all other components both inside and outside the fence, while enclosure cultivation significantly increased the biomass of the roots, leaves, and spikes. These observations may be attributable to the fact that in arid desert steppes, strengthening root growth is one of the most effective strategies for plants to obtain moisture and inorganic nutrients for growth and reproduction (Zhou, Yan, Xiao, Wang, & Kuang, 2015). Second, these changes may be due to the use of enclosures, which markedly increases the number of effective tillers in *A. mongolicum*, inducing a significant increase in the biomass of the roots, leaves, and spikes. In addition, the root biomass of *A. mongolicum* inside the fence significantly increased, which may be a result of satisfying the higher nutrient and water requirements of *A. mongolicum* after enclosure.

Carbon, nitrogen, and phosphorus are macronutrients that are essential to plant growth and reproduction and can reflect plant nutrient limitation status at the population (Gallardo & Covelo, 2005), community (Güsewell, 2004), ecosystem (Tessier & Raynal, 2003), and global (Han, Fang, Guo, & Zhang, 2005) levels. Xu et al. (2014) conducted studies on stoichiometric changes in elements in plants subjected to different utilization methods in the Inner Mongolian temperate grasslands. The results showed that there were no significant changes in total carbon content in the leaves, whereas total nitrogen content significantly decreased in plants in enclosed conditions. In this study, the roots showed the lowest total carbon, nitrogen, and phosphorus contents, whereas the spikes exhibited the highest contents. After enclosure cultivation, no significant changes in total carbon and phosphorus levels were observed in the leaves, whereas total nitrogen content significantly decreased. These results agree with the conclusions of the aforementioned studies (Xu et al., 2014). In addition, these observations show that the enrichment and allocation of elemental nutrients in nonreproductive (branches and leaves) and reproductive (flowers and fruits) organs is an evolutionary response of plant populations toward specific environments. The elemental nutrient composition of plants is in stable dynamic equilibrium. When changes in external environmental conditions (enclosure cultivation) occur, plants undergo self‐adjustment to maintain stability in elemental composition (Zhao, 2007).

The composition of fats, proteins, and carbohydrates varies among plant components. Therefore, there are large differences in caloric values among components. The reproductive allocation status of energy in plant populations can reflect the degree of adaptability of a certain species to the environment and the limitation status of environmental resources on population growth (Wang, 2000). In this study, the caloric values of *A. mongolicum* components inside and outside the fence showed a decreasing trend of spikes > leaves > stems > roots, and the mean caloric values of aboveground organs were all greater than that of underground roots. This result agrees with the findings of previous studies (Bao et al., 2006; Jiang, Fan, Ge, & Tian, 2014). Components with greater fat and protein content have higher caloric values, whereas components with higher carbohydrate content have lower caloric values (Jiang et al., 2014). Leaves and other photosynthetic organs can convert solar energy to chemical energy that can to be stored in plant tissues. This is also an important reason why leaves have higher caloric value (Bao et al., 2006). In addition, the caloric values of spikes and roots significantly increased, and the caloric value of leaves significantly decreased after enclosure cultivation. This shows that after enclosure cultivation, good habitat increases energy in *A. mongolicum* populations, enabling more caloric values to be allocated to reproductive components to increase reproduction and growth (Zhao, 2007). Additionally, root caloric values increase to satisfy the higher nutrient requirements of the population.

Carbon, nitrogen, and phosphorus are major intrinsic factors that affect plant caloric values, and there are studies that have focused on the effects of carbon and nitrogen on caloric values (Ren & Peng, 1999). Gao, Xie, Xu, and Han (2012) studied the caloric values of 15 plants in the *Aneurotepidimu chinense* grassland in the Xilin River Basin in Inner Mongolia. Their results indicated significant positive correlations between total carbon and total nitrogen content and caloric values. In this study, the caloric values of *A. mongolicum* showed significant positive correlations with total carbon, nitrogen, and phosphorus, which is consistent with the results of previous studies (Gao et al., 2012; Guo, Huang, Chao, & Zhu, 2005; Xu et al., 2003). Carbon is a major substance that is combusted by plants that directly affects plant caloric value. Similarly, nitrogen content can indirectly affect plant caloric value during combustion in plants.

## 5 CONCLUSIONS

Enclosure cultivation can significantly increase the height, clump width, coverage and individual clump biomass of *A. mongolicum*, but not density. After enclosure cultivation, the biomass of the roots, leaves, and spikes of *A. mongolicum* populations significantly increased. Enclosure cultivation was found to have varying degrees of effects on elemental nutrients and caloric values of vegetative and reproductive components of *A. mongolicum*, and the caloric values of the *A. mongolicum* components were significantly positively correlated with carbon, nitrogen, and phosphorus contents. These findings indicate that the enrichment and allocation of elemental nutrients and caloric values in nonreproductive (stems and leaves) and reproductive (spikes and seeds) organs is an evolutionary response of plant populations toward specific environments.

## CONFLICT OF INTEREST

The authors declare that they have no conflict of interest.

## AUTHOR CONTRIBUTIONS

GQL conceived and designed the experiment; PPZ and WSS performed materials and data analysis; PPZ and GQL wrote the paper; PPZ, WSS, CQJ, LXS and YYC carried out field investigations.

## Data Availability

When accepted, all data will be uploaded and archived in Dryad (Dryad https://doi.org/10.5521/dryad.12311).
